# Telehealth in the COVID-19 Era: A Balancing Act to Avoid Harm

**DOI:** 10.2196/24785

**Published:** 2021-02-01

**Authors:** J Jeffery Reeves, John W Ayers, Christopher A Longhurst

**Affiliations:** 1 Department of Surgery University of California San Diego La Jolla, CA United States; 2 Department of Medicine Division of Biomedical Informatics University of California San Diego La Jolla, CA United States

**Keywords:** telehealth, patient safety, COVID-19, coronavirus, informatics, safety, harm, risk, access, efficiency, virtual care

## Abstract

The telehealth revolution in response to COVID-19 has increased essential health care access during an unprecedented public health crisis. However, virtual patient care can also limit the patient-provider relationship, quality of examination, efficiency of health care delivery, and overall quality of care. As we witness the most rapidly adopted medical trend in modern history, clinicians are beginning to comprehend the many possibilities of telehealth, but its limitations also need to be understood. As outcomes are studied and federal regulations reconsidered, it is important to be precise in the virtual patient encounter approach. Herein, we offer some simple guidelines that could assist health care providers and clinic schedulers in determining the appropriateness of a telehealth visit by considering visit types, patient characteristics, and chief complaint or disease states.

## Introduction

The telehealth revolution has been heralded for its potential to increase health care access and improve the efficiency of health care delivery. Previously, telehealth had been strategically rolled out to settings wherein the need for a relationship is less pressing (eg, radiology), outcomes can be remotely assessed in principle (eg, dermatology), or access to care is severely limited (eg, rural areas) [1]. COVID-19 prompted a near-universal expansion of telehealth utilization. Many applaud this change [2]. However, virtual patient care can bring unintended consequences that eclipse its benefits, including potentially limiting the patient-provider relationship, quality of examination, the efficiency of health care delivery, and overall quality of care [3-6]. As safety protocols for in-person evaluation have been developed, critical examination raises serious dilemmas about the harms of telehealth that can inform a new agenda for how to best utilize telehealth in the COVID-19 era. As we witness the most rapidly adopted medical trend in modern history, clinicians are beginning to comprehend the possibilities of telehealth, but its limitations also need to be understood.

## The Rise of Telehealth in Response to COVID-19

Around the globe, patients are wary of entering health care settings, and providers fear unnecessary exposure for both patients and themselves, thereby creating a familiar dilemma of an unprecedented variety: How to best care for patients while *first doing no harm?*

In response, health systems have rapidly transitioned patient care away from in-person encounters towards telehealth [7]. This transition was facilitated by legislative changes designed to enable the “good faith” provision of health care at a distance, thereby limiting COVID-19 transmission [8]. New reimbursement policies have expanded access to a range of telehealth services offered by medical providers, clinical psychologists, licensed clinical social workers, and other health care workers.

In a matter of weeks, the application of patient-facing technology spread across all outpatient settings, including primary and preventative, medical and surgical specialty, and mental health care. Equipped with remote monitoring, access to multi-provider video visits, and virtual translators, some specialty clinics adopted a 100% virtual approach. Telehealth found its way into emergency departments, hospital wards, intensive care units, and even interdisciplinary services such as occupational and physical therapy [9]. Telehealth was originally regarded as a form of health care delivery largely reserved for specific, resource-limited settings, but it is now embedded in the daily practice of providers across the spectrum of patient care.

## Unintended Consequences of Telehealth

First, the most obvious potential harm of telehealth is an incomplete or inaccurate physical examination. Advances in patients’ technological assessments have historically led to the abandonment of physical touch and examination in general—COVID-19 potentiated this issue [10]. Several reports have documented the steps to perform a virtual physical examination that can be applied to a wide range of patient encounters [11]. For example, Tanaka et al [12] describe creative ways of performing a virtual orthopedic examination. However, there are real limitations to a virtual examination, including the potential requirement of assistance from a caregiver, inadequate patient-home environment or space, poor lighting or discoloration with resultant poor visualization, increased time required to perform an assessment, and other technical issues [6,13,14]. In particular, the assessment of a patient’s movement and motor function is limited, in part due to the need for a patient-assisted examination that can be challenging to capture on video [6,15,16]. Other aspects of a physical examination simply cannot be done via telehealth, such as auscultation of heart or lung sounds [17]. Thus, the true effectiveness of a telehealth physical examination and its subsequent impact on diagnosis, clinical management, and medical outcomes is yet to be determined in all settings.

In some instances, health care providers have abandoned the physical aspect of the “history and physical.” The Centers for Medicare and Medicaid Services reimburse for telehealth annual well visits—an encounter that historically offered providers an opportunity to perform a thorough examination for subtle abnormalities is now often performed purely on the telephone. In other settings, reliance on technology has replaced physical examination. Inpatient cardiology consults are now performed by providers using apps on their devices (eg, iPhones and iPads) that allow real-time electrocardiography with an acceptable neglect of a careful, in-person examination of neck veins and body tissue to assess fluid status.

Beyond the clinical value, many patients feel comforted and reassured after a physical examination. Others perceive a virtual examination as inadequate regardless of its true quality [18]. “We must not forget the importance of a doctor’s hands” [19]—a phrase that used to symbolize compassion is now, based on Google search results, associated with COVID-19 safety information and online advice to self-isolate.

Second, the roll-out of telehealth undermines the patient-provider relationship and the essential humanistic qualities of care providers. Although the world has become accustomed to online relationships, a trusting and personal connection between the patient and the health care provider, a vital aspect of health care, can be challenging to build with purely digital interaction. Multiple patient experience studies have consistently reported difficulties in communicating or connecting with providers during telehealth visits [3,6]. A desire for building an improved rapport with their providers is one of the most popular reasons why many patients prefer in-person visits [20]. In particular, the establishment of primary care or the initial consultation, similar to a first date, may not be welcomed by all and could negatively affect patient experience or the development of a sense of “my doctor.” A survey of primary care patients during the pandemic found that telehealth was considered the most appropriate in the presence of a pre-existing clinical relationship [21]. An analysis of 620 patient satisfaction survey outcomes from clinic encounters found that new visit type was associated with lower patient satisfaction (parameter estimate -0.75; 95% CI -1.00 to -0.049) [22]. Consider the impact on the patient-centered care model, in which empathy, two-way communication, and direct eye-to-eye contact are considered crucial elements to improving health literacy and engaging patients in their own disease management [23]. Depending on the situation, telehealth may add one more barrier to understanding complex medical disease and patient compliance.

Third, the expansion of virtual services to rates beyond what was previously expected has resulted in inefficiencies in health care delivery. A mass of health care organizations rapidly transitioned and onboarded providers to telehealth encounters in a matter of weeks during the early-stage of the COVID-19 pandemic [24]. Although telehealth can provide thorough, safe, and effective medical care remotely, there is a learning curve associated with its implementation, and its success requires logistics that are frequently overlooked. For instance, clinics have not been appropriately restructured to support telehealth visits, resulting in under-utilization of talent. For example, medical assistants now play a role similar to that of front-desk personnel, thereby considerably limiting their participation in patient care. As such, the existing infrastructure, education, and administrative support surrounding telehealth must be tailored and broadened.

## Safety of Health Care Facilities

The rapid expansion of telehealth was intended to protect uninfected patients from the risks of COVID-19 during periods of uncertainty. Although disease transmission is known to occur in medical facilities, growing evidence suggests that health care workers are more likely to be exposed to the virus while performing extra-occupational activities [25,26]. More experience in handling COVID-19 cases, coupled with an increasing knowledge base of its characteristics and modes of transmission, has led to the establishment of evidence-based protocols proven to facilitate the safe delivery of traditional brick-and-mortar patient care [27,28]. Retrospective studies have shown that transmission of the virus among health care workers is considerably reduced when these protocols are followed [29]. Proper hand hygiene, facial coverings, use of appropriate personal protective equipment, and implementation of social distancing practices allow the safe return of routine in-person services [30]. Additionally, the redesign of clinic spaces and the use of electronic registration with automated messaging to avoid crowded waiting areas can further enhance the safety of in-person patient encounters. As such, providers now can be more precise in which patients are seen physically rather than virtually.

## Updating Telehealth for the COVID-19 Era

For certain encounters, it makes intuitive sense to conduct telehealth visits remotely, and this may ultimately become the standard of care in the wake of the COVID-19 pandemic. For example, during a medication review for patients with chronic medical conditions, a physician can ask the patient to point the camera to the labels of the pills they are actually taking. On the other hand, some chief complaints require a physical encounter—a newly recognized palpable mass, for instance. The problem lies in the equivocal encounters: the patient who keeps physicians up at night, making them wonder if they should have recommended a trip to the dreaded emergency department, or the well-child, who is up to date on immunizations but has not had their weight or development assessed. As clinics reopen, finding the optimal balance of in-person and telehealth patient encounters is one of the most critical questions facing health care delivery. Appropriateness of telehealth visits likely varies according to the type of service being offered, medical specialty, health system, and geographic location.

We offer some simple guidelines to consider in determining the appropriateness of a telehealth visit (Table 1); these guidelines may be utilized by health care providers and, importantly, clinic schedulers. First, the visit type should be considered. New patient visits, even those that perhaps are safe to perform virtually, may benefit from face-to-face encounters to familiarize patients with their care and build a trusting relationship. Follow-up visits or new patients unlikely to need a physical examination may be appropriately managed via telehealth. Second, many patient characteristics are important to be considered, including health literacy, the structure of social support systems, hearing ability or visual acuity, and simple preference. A simple assessment of these 2 factors can likely be made by clinic schedulers with no further context of a patient’s medical history. Thus, appropriate delineation of telehealth utilization in the outpatient setting can be made primarily without the consultation of a provider. However, for borderline patients, a provider can consider the chief complaint and chronicity of disease state as key determinants of whether in-person care with a highly reliable physical examination is necessary. When a physical examination may change either the diagnosis or management strategy, the patient should be seen in-person. This distinction can often be determined based on the chief complaint [31]. Finally, the technological capabilities of the patient must not be overlooked or taken for granted.

In the future, precision in the approach and delivery of the telehealth patient encounter is essential. The telehealth revolution has been impressive and has made substantial contributions to the global management of this pandemic, but significant work remains to ensure the best health care delivery for our patients. We have an opportunity to simultaneously embrace the necessity and benefits of telehealth while supporting strategies that optimize patient outcomes and humanism in medicine. Universally, health care workers have labored under extraordinary circumstances with incredible and heart-warming fortitude. Front-line providers have reminded us of the virtue of health care. There is no certainty about what the postpandemic world will look like. However, there will always be patients in need, and it is our responsibility to ensure they receive the high-quality physical and personal care they deserve.

**Table 1:** Characteristics to consider for determining the appropriateness of telehealth.

Characteristic	Appropriate for telehealth	Potentially inappropriate for telehealth
Visit type	Follow-up visit for known/diagnosed disease state or patient condition	New patient (establishment of primary care or new consultation)
	Follow-up postprocedure visit with no patient complaints	Annual physical examination or well-child check
	Recurring medication or chronic medical condition review	Acute visit prompted by an acute change in patient condition
	Initial or follow-up visit for mental health conditions	Initial psychiatry visits or annual follow-up for controlled substances
Patient characteristics	Existing, trusting, personal connection with the provider	Distrusting of health care professionals
	High health literacy	Low health literacy
	Robust social support system	Low social support system
	Anxious in health care settings	Poor vision or hearing
	Lives remotely or has inadequate transportation	Prefers in-person
	Prefers telehealth	
Chief complaint or disease state characteristics	Physical examination unlikely to be diagnostic	Physical examination may aid in diagnosis or prognosis
	N/A^a^	Examination findings may influence initial workup and/or management
	Focused physical examination can be performed virtually (ie, visual examination)	Focused examination cannot be performed virtually (ie, palpation of mass)
	Chief complaint with standardized initial workup and management	Chief complaints that often result in referral to acute care settings
Other considerations	English as a second language (interpreter required)	Patient has a poor internet connection
	Patient or close family member technologically savvy	Patient lacks technological capabilities to join video visit/telehealth encounter
	Patient with multiple family members at home who can join telehealth visit	N/A

^a^N/A: not applicable.

## References

[1] Moore MA, Coffman M, Jetty A, Klink K, Petterson S, Bazemore A (2017). Family physicians report considerable interest in, but limited use of, telehealth services. J Am Board Fam Med.

[2] Mehrotra A, Ray K, Brockmeyer D, Barnett M, Bender J (2020). Rapidly converting to “virtual practices”: outpatient care in the era of Covid-19. NEJM Catal Innov Care Deliv.

[3] Mair F, Whitten P (2000). Systematic review of studies of patient satisfaction with telemedicine. BMJ.

[4] Wachter R (2016). The digital doctor: hope, hype, and harm at the dawn of medicine’s computer age. Crit Care Nurse.

[5] Dorsey ER, Topol EJ (2016). State of telehealth. N Engl J Med.

[6] Saliba-Gustafsson EA, Miller-Kuhlmann R, Kling SMR, Garvert DW, Brown-Johnson CG, Lestoquoy AS, Verano M, Yang L, Falco-Walter J, Shaw JG, Asch SM, Gold CA, Winget M (2020). Rapid implementation of video visits in neurology during COVID-19: mixed methods evaluation. J Med Internet Res.

[7] Reeves JJ, Hollandsworth HM, Torriani FJ, Taplitz R, Abeles S, Tai-Seale M, Millen M, Clay BJ, Longhurst CA (2020). Rapid response to COVID-19: health informatics support for outbreak management in an academic health system. J Am Med Inform Assoc.

[8] Shachar C, Engel J, Elwyn G (2020). Implications for telehealth in a postpandemic future: regulatory and privacy issues. JAMA.

[9] Keesara S, Jonas A, Schulman K (2020). Covid-19 and health care’s digital revolution. N Engl J Med.

[10] Horton R (2019). Offline: touch—the first language. The Lancet.

[11] Benziger CP, Huffman MD, Sweis RN, Stone NJ (2021). The telehealth ten: a guide for a patient-assisted virtual physical examination. Am J Med.

[12] Tanaka MJ, Oh LS, Martin SD, Berkson EM (2020). Telemedicine in the era of COVID-19: the virtual orthopaedic examination. J Bone Joint Surg Am.

[13] Mammen JR, Elson MJ, Java JJ, Beck CA, Beran DB, Biglan KM, Boyd CM, Schmidt PN, Simone R, Willis AW, Dorsey ER (2018). Patient and physician perceptions of virtual visits for Parkinson's disease: a qualitative study. Telemed J E Health.

[14] Kemp MT, Liesman DR, Williams AM, Brown CS, Iancu AM, Wakam GK, Biesterveld BE, Alam HB (2020). Surgery provider perceptions on telehealth visits during the COVID-19 pandemic: room for improvement. J Surg Res.

[15] Hoenig H, Tate L, Dumbleton S, Montgomery C, Morgan M, Landerman LR, Caves K (2013). A quality assurance study on the accuracy of measuring physical function under current conditions for use of clinical video telehealth. Arch Phys Med Rehabil.

[16] Yager PH, Clark ME, Dapul HR, Murphy S, Zheng H, Noviski N (2014). Reliability of circulatory and neurologic examination by telemedicine in a pediatric intensive care unit. J Pediatr.

[17] Chowdhury D, Hope KD, Arthur LC, Weinberger SM, Ronai C, Johnson JN, Snyder CS (2020). Telehealth for pediatric cardiology practitioners in the time of COVID-19. Pediatr Cardiol.

[18] Powell RE, Henstenburg JM, Cooper G, Hollander JE, Rising KL (2017). Patient perceptions of telehealth primary care video visits. Ann Fam Med.

[19] Verghese A (2008). Culture shock — patient as icon, icon as patient. N Engl J Med.

[20] Lacritz L, Carlew A, Livingstone J, Bailey K, Parker A, Diaz A (2020). Patient satisfaction with telephone neuropsychological assessment. Arch Clin Neuropsychol.

[21] Imlach F, McKinlay E, Middleton L, Kennedy J, Pledger M, Russell L, Churchward M, Cumming J, McBride-Henry K (2020). Telehealth consultations in general practice during a pandemic lockdown: survey and interviews on patient experiences and preferences. BMC Fam Pract.

[22] Ramaswamy A, Yu M, Drangsholt S, Ng E, Culligan PJ, Schlegel PN, Hu JC (2020). Patient satisfaction with telemedicine during the COVID-19 pandemic: retrospective cohort study. J Med Internet Res.

[23] (2017). What Is Patient-Centered Care? NEJM Catal. NEJM Catalyst.

[24] Patel PD, Cobb J, Wright D, Turer R, Jordan T, Humphrey A, Kepner AL, Smith G, Rosenbloom ST (2020). Rapid development of telehealth capabilities within pediatric patient portal infrastructure for COVID-19 care: barriers, solutions, results. J Am Med Inform Assoc.

[25] Lan F, Wei C, Hsu Y, Christiani DC, Kales SN (2020). Work-related COVID-19 transmission in six Asian countries/areas: A follow-up study. PLoS One.

[26] Lentz RJ, Colt H, Chen H, Cordovilla R, Popevic S, Tahura S, Candoli P, Tomassetti S, Meachery GJ, Cohen BP, Harris BD, Talbot TR, Maldonado F (2020). Assessing coronavirus disease 2019 (COVID-19) transmission to healthcare personnel: The global ACT-HCP case-control study. Infect Control Hosp Epidemiol.

[27] Centers for Disease ControlPrevention Following Infection Prevention and Control Recommendations in Healthcare Settings During the COVID-19 Pandemic. Healthcare Facilities: Managing Operations During the COVID-19 Pandemic.

[28] Gan WH, Lim JW, Koh D (2020). Preventing intra-hospital infection and transmission of coronavirus disease 2019 in health-care workers. Saf Health Work.

[29] (2020). Coronavirus disease 2019 (COVID-19): situation report, 82. World Health Organization.

[30] (2020). Interim Infection Prevention and Control Recommendations for Healthcare Personnel During the Coronavirus Disease 2019 (COVID-19) Pandemic. Centers for Disease Control and Prevention - COVID-19.

[31] Gadzinski AJ, Ellimoottil C (2020). Telehealth in urology after the COVID-19 pandemic. Nat Rev Urol.

